# Viral Factors in Non-Progression

**DOI:** 10.3389/fimmu.2013.00355

**Published:** 2013-12-24

**Authors:** Bin Wang

**Affiliations:** ^1^Sydney Medical School, University of Sydney, Sydney, NSW, Australia

**Keywords:** HIV, accessory genes, genetic mutation, non-progressive disease, Nef, vpr

## Abstract

Research has undergone considerable development in understanding a small subset of human immunodeficiency virus type 1 (HIV-1)-infected, therapy-naive individuals who maintain a favorable course of infection surviving for longer periods of time. Although, viral, host genetic, and immunological factors have been analyzed in many previous studies in order to delineate mechanisms that contribute to non-progressive HIV disease, there appears to be a no clear cut winner and the non-progressive HIV disease in <1% of HIV-infected individuals appears to be a complex interplay between viral and host factors. Therefore, it is important to review them separately to signify their potential contribution to non-progressive HIV disease. With respect to virological features, genomic sequencing of HIV-1 strains derived from long-term non-progressors has shown that some individuals are infected with attenuated strains of HIV-1 and harbor mutations from single nucleotide polymorphisms to large deletions in HIV-1 structure, regulatory, and accessory genes. The elucidation of functional attributes of defective/attenuated HIV strains may provide better understanding of viral pathogenesis and the discovery of new therapeutic strategies against HIV. This review mainly focuses on the defects in viral genes that possibly guide non-progressive HIV disease.

## Introduction

Since the beginning of the AIDS epidemic in 1980s’, scientists have made great efforts to understand the nature of human immunodeficiency virus (HIV) disease and of its causal agent, the HIV. After primary human immunodeficiency virus type 1 (HIV-1) infection, majority of infected individuals display a gradual decline in peripheral blood CD4^+^ T lymphocytes throughout the course of the illness accompanied by progressive loss of protective immunity against pathogens (1). However, the natural course of HIV infection such as the progression rates to AIDS and clinical manifestations of diseases associated with infection differs greatly. About 1% of HIV-1^+^ patients are able to maintain stable CD4^+^ T-cell counts within the normal range for a prolonged period of time and remain asymptomatic without anti-retroviral therapy (ART). These HIV-1 infected asymptomatic individuals are often referred to as long-term non-progressors (LTNPs). Within this group, a subset of individuals shows plasma HIV-RNA values persistently below detectable level (50 copies/ml), and termed “elite” or “natural controllers” (EC) (2). Study of mechanisms that contribute to non-progressive HIV disease have revealed complex interplay between viral and host factors. In this section, viral genomic features that associated with benign course of HIV infection will be discussed to delineate our understanding of viral factor in non-progression.

## HIV-1 Genomic Attenuation that Contribute to Non-Progressive HIV Disease

Human immunodeficiency virus type 1 genome and proteins has been the subject of extensive research since its discovery in 1983 (3). Among nine genes coded by HIV-1, three genes, gag, pol, and env, are found in all retroviruses and are essential to make structural proteins. The other six genes, tat, rev, nef, vif, vpr, and vpu, often described as regulatory and accessory genes, code for proteins that are unique to HIV with important roles on the viral replication and the development of AIDS through many complicated mechanisms. Early identifications of viral attenuation *in vitro* (4, 5) coupled with the observation of low viral loads *in vivo* and decreased disease progression rate point to the possibility that viruses present in these individuals may be attenuated or defective. Extensive analysis of the HIV-1 genomes, particularly in the regulatory/accessory genes, has shown that certain genetic defects may confer protection to the host.

## Accessory Gene Attenuation and Disease Progression

Although initial thought to be dispensable for infection, HIV-1 accessory proteins have now been considered to be important factors that determine the replication and pathogenesis for efficient infection *in vivo*.

### Nef

Nef has emerged as one of the most important proteins for viral life cycle and pathogenesis. This accessory protein exhibits a spectrum of biological activities including down-regulation of human leukocyte antigen class I (HLA-I), down-regulation of CD4, enhancement of virion infectivity, and stimulation of viral replication (6–11). Infection by HIV-1 with truncated nef has been shown to contribute to low-level virus replication and non-pathogenicity (12–14). This was further supported by animal study of macaques infected by a nef-deleted SIVmac239 that displayed the absence of disease progression and maintained greatly reduced viral load (15). However, subsequent study using nef-deleted SIVmac239 as live attenuated vaccine fail to demonstrate the safety and efficacy in neonatal macaques (16). In addition to large deletions in nef, single amino acid substitution via point mutation that impairs the viral fitness and replication were also reported to slow the disease progression. The detection of significant increased incidence of single amino acid polymorphism at position 138 in LTNPs/SPs (17–19) and the discovery of 40% of HIV-infected children who experiencing delay disease progression carrying amino acid substitutions at the AWLEAQ (56–61) and the Rxx (22–24) domain responsible for the abolishing of CD4 and MHC-1 down-regulations (20) demonstrated functionally defective nef HIV-1 can be raised without gross gene deletion (Table 1). Mutation studies from several groups have further identified residues involved in nef biological activities such as residues of R25, RD35/36, T80, GL96/97, D108, D111, DW123/124, RY134/135, C142, EE154/155, LL164/165, DD174/175, RRE179, RF184/185 participated in the CD4 down regulation (21–25). Genetic mutations occurred within these residues may potentially disrupt Nef functions and contribute to non-progressive HIV infections.

**Table 1:** **Summary of HIV-1 genetic mutations that associated with non-progressive HIV disease**.

		Locations	Functional changes	Reference
Structure genes	gag	S67A and D102E	No functional support	Miura et al. (26)
		Single and double amino acid deletions in gag’s p17 and p6	No functional support	Alexander et al. (27)
		Stop codons in the gag p17 and p27	No functional support	Wang et al. (28)
	
	pol	M184V/I	Reduced replication capacity	Harrison et al. (29)
	
	env	V2 loop extension	Restrict the capacity of HIV-1 to replicate in macrophages	Shioda et al. (30), Wang et al. (31)
		Single amino acid deletion in gp41	Reduced replication capacity	Alexander et al. (27)

Regulatory genes	tat	HIV-1OYl; substitution of cysteine residue for a serine	Unable to *trans-*activate	Huet et al. (32)
	
	rev	Three amino acids extension at the 3′end of rev exon 2	No functional support	Papathanasopoulos et al. (33)
		Polymorphism of codon 78 (L78I)	Reduce the export of Rev from the nucleus to the cytoplasm	Iversen et al. (34)
		Glu74Pro, Val 104 Leu, and Ser 106 Pro	RRE binding ability	Churchill et al. (35)

Accessory genes	vpr	Amino acid substitutions at position 72 (F72L)	Reduce nuclear accumulation and decrease incorporation of vpr into the forming virions	Caly et al. (36)
		Amino acid substitutions at position 77 (R77Q)	Reduce cytopathicity	Lum et al. (37), Mologni et al. (38)
		C-terminus amino acid deletions 83–89	Defective in nuclear localization; lost ability to induce G2 arrest and cell death	Wang et al. (39), Zhao et al. (40)
	
	vif	195 nucleotides deletion (aa54–117), insertion in position 63, premature stop codons at positions 70 and 174	No functional support	Rangel et al. (41)
		V13I, V55T, and L81M	No functional support	De Maio et al. (42)
		Amino acid substitutions at position (R132S)	Reduced replication capacity	Hassaine et al. (43), Fujita et al. (44)
	
	vpu	Four-amino-acid insertion in the N terminus	No functional support	Alexander et al. (27)
	
	nef	160–430 nucleotides deletion in nef-LTR region	Low-level virus replication and reduced pathogenicity	Deacon et al. (12)
		109–139 nucleotides deletion in nef gene and 159–204 deletion in nef-LTR region	No functional support	Salvi et al. (13)
		84 to >400 bp nucleotides deletion in nef-LTR region (CRF01_AE)	No functional support	Kondo et al. (14)
		Amino acid polymorphism of at position 138 (LTNP/SP)	Decreased viral replication	Premkumar et al. (17), Kirchhoff et al. (18); Tolstrup et al. (19)
		Amino acid substitutions at position 22–24 or 56–61	Abolishing nef mediated CD4 and MHC-1 down-regulations	Corro et al. (20)

### VPR

The viral protein R (vpr) of HIV-1 is a highly conserved small basic protein and contributes to viral replication and disease progression *in vivo*. vpr Functions include G2 cell cycle arrest and apoptosis, T-cell depletion, and nuclear localization of the HIV preintegration complex (36, 37, 45). vpr Also plays a critical role in long-term AIDS disease by inducing infection in non-dividing cells such as macrophages (46). Functional analysis of vpr protein has provided insights into the biological role played by this protein during the virus life cycle (47–49) and also implied the mutations potentially affect vpr functions. The phenylalanine to leucine mutation at amino acid position 72 (F72L) detected from a non-progressor has been shown to reduce nuclear accumulation and decrease incorporation of vpr into the forming virions (36), while R77Q mutations at the C-terminal conserved motif between 71 and 82 has the ability to reduce cytopathicity and are more frequently detected in LTNPs (37, 38). This mutation may also interfere with vpr-mediated cell cycle arrest (Table 1) (37, 38, 50). The gross defect of vpr gene was uncommon and comparative study of long-term asymptomatics and progressors often showed full-length and intact open reading frames (51) and only an early study reported vpr gene defects clustered at the C-terminus (amino acid 83–89) in long-term non-progressing mother child pair that may potentially affect its secondary structure (39). Subsequent functional analyses of these naturally occurring C-terminus polymorphisms have indicated defective in their ability to localize onto the nuclear envelope, lost ability to induce G2 arrest and lost the ability to induce cell death in some of the clones (Table 1) (40).

### Vpu

The Vpu is a transmembrane protein with a key function in interacting with newly synthesized CD4 molecule in the rough endoplasmic reticulum (RER) resulting in its degradation via the proteasome pathway (52, 53). The other functions of Vpu include enhancement of virion release from virus-producer cells and down-regulation of MHC I and II (54–57).

There is very limited data available on genetic defects in the vpu gene and its association with disease progression as most studies revealed the absence of gross deletions or insertions in the Vpu derived from LTNPs (51, 58). So far, only one non-progressive individual with a four-amino-acid insertion in the N terminus of Vpu was reported (27). However, the presence of 4-bp insertion in *nef* and 3′-LTR sequences resulting the truncation of Nef by one amino acid short of consensus C-terminal cysteine in the same individual made it difficult to determine the significance of Vpu contribution to the non-progressive status. Interestingly, although there is insufficient patient derived data on Vpu defects and disease progression, it was proposed that lack expression of a functional Vpu protein, such as HIV-2 and most SIV isolates, may be responsible for slower disease progression and cause less disease severity (59).

### Vif

The HIV-1 Vif protein (virion infectivity factor) has a essential role in promoting HIV-1 infectivity by enhancing viral replication and inducing the degradation of the endogenous anti-retroviral factor, apolipoprotein B mRNA editing enzyme catalytic polypeptide- like 3G (APOBEC3G) (60, 61). The importance of vif gene has been well recognized, but only few polymorphisms have been described in possible association with a retarded progression to AIDS. After sequencing vif and nef gene from 14 slow progressors and 46 normal progressors, Rangel et al. revealed the co-circulating of intact and truncated vif gene in one slow progressor. In the same study, the presence of amino acid insertion at position 63 and premature stop codon were also observed in two other slow progressors. But the detection of stop codon in the vif gene in a normal progressive patient with high viral load also suggesting such inhibitory mutations in the vif gene may be less important in virus load reduction (41). A very recent study of a group of 11 children with an extremely slow disease progression found unusual substitutions such as V13I, V55T, and L81M. Databases search suggested an increased frequency of these mutations in sequences from elite controllers (42). Whether these changes linked to Vif functional alternation require further investigations. In addition, R132S substitution has been described to present in LTNP and SP with *in vitro* evidence of reduced viral replication (43, 44). In contrast, one amino acid insertion at position 61 and the substitutions of A62D/N/S and Q136P was indicated to be associated with an accelerated AIDS outcome (62).

## Regulatory Gene Attenuation and HIV Disease Progression

Tat (*T*rans-*A*ctivator of *T*ranscription) and Rev (*Re*gulator of *V*irion protein expression) are two essential viral regulatory factors to promote high levels of viral gene expression (63–66). Duo to the function importance, defective HIV-1 rev and tat gene are rarely reported. HIV-1 Tat promotes efficient transcription of the viral genome, which requires structural changes of Tat to bind to a RNA stem-loop structure called TAR (transactivation response element) (67, 68). Study of an unusual HIV-1 strain isolated from a healthy Gabonese individual who presented an atypical western blot has revealed functionally defective of Tat resulting from the substitution of an essential cysteine residue for a serine (32). Although the defected Tat has a similar structure to active Tat, it is unable to *trans-*activate (69). This virus, identified as HIV-1OYl, grew to low titers of reverse transcriptase activity, and is lack of obvious cytopathic effect. Important to note that 10 years post infection, 23 of HIV-1 OY1 infected women showed retro conversion and HIV was no longer detectable (70).

In HIV-1 Rev, early study by Iversen et al. (34) revealed the polymorphism of codon 78 (L78I) in the activation domain might contribute to non-progression status (Table 1). Substitutions in this highly conserved leucine-rich activation domain are known to reduce the export of Rev from the nucleus to the cytoplasm (34, 71) and associate with decrease in viremia (72). A three amino acids extension (GlyCysCys) at the 3′ end of rev exon 2 instead of characteristic 16-amino acid truncation commonly shared by HIV subtype C was also reported in HIV-1 subtype C infected slow-progressing siblings (33). In well-characterized Sydney Blood Bank Cohort, in addition to nef attenuation, several members have also displayed functional defect in Rev by evaluation of RRE binding ability (35). Three rare amino acid changes at highly conserved residues (Glu 74 Pro, Val 104 Leu, and Ser 106 Pro) were likely to be associated with such functional defect in two of the cohort’s members (Table 1) (35).

## Structural Genes and HIV Disease Progression

*HIV-1 env gene* (gp160) product consists of two subunits, gp120 and gp41, and play a crucial role in viral infectivity by binding to CD4 and chemokine receptors expressed on the surface of susceptible cells. The chemokine receptors usage, generally CCR5 and/or CXCR4, are determined largely by amino acid sequence of the variable loop 3 (V3) of gp120 (73). CCR5-using viruses (R5 viruses) are presence in the vast majority of primary infections while a receptor switch toward CXCR4 occurs in about 50% of the infected individuals which is associated with increasing in viral load, accelerated CD4^+^ T-cell decline and progression to AIDS (74–76). Therefore, the coreceptor switch could be a key element of HIV pathogenesis and a significant contribution to disease progression. However, the reasons for the coreceptor switch remain poorly understood (77). In LTNPs cohort, HIV-1 strains isolated displayed not only the exclusive CCR5 usage but also decreased entry efficiency suggesting lower env fitness in LTNPs cohort that may contribute to viral suppression (78).

Apart from V3 loop, the sequence changes in the first and second hypervariable loops (V1 and V2) also affect the viral phenotypic property and cellular host range. Several independent studies have shown V2 loop extension in individuals with slow or no disease progression (Table 1) (30, 31). This elongation of V2 may potentially restrict the capacity of HIV-1 to replicate in macrophages (30).

In comparison to HIV-1 gp120, the mutations in the fusion protein subunit gp41 were less frequently reported in their influence in the disease progression rate. A single amino acid deletion in the fusion peptide region of the transmembrane domain in one LTNP was speculated to be responsible for the slow/low growth phenotype of the virus isolated from this individual (27).

### Gag and Pol

Miura et al. studied viral gag sequences from 50 non-progressions and 80 progressors revealed three codon changes (67A, 102E, and 389I) that were significantly different between the two groups (26). Two of the three codons, S67A and D102E, showed a strong association with the non-progressive HIV disease. However, recombinant viruses with these two mutations failed to provide evidence on the impact of viral replication capacity indicating these differences may merely reflect the historic population consensus amino acid at the time of infection (Table 1) (26). Similarly, functional study of single and double amino acid deletions observed in gag’s p17 and p6 from 5 of the LTNPs revealed no difference in facilitating the incorporation of vpr into the HIV-1 particles (27). Apart from the sequence polymorphisms, stop codons in the gag p17, p27, and in pol RT in proviral DNA from one LTNP have also been reported as a consequence of G-A hypermutation (28). The highly homogeneous sequences with the inactive mutations over 8-year period in this individual suggesting only limited proviral integration events occurred. However, the detection of persist antibody responses to both p17 and p24 proteins by western blot during the same period suggesting the presence of intact virions during the course of infection and possible persistent viral replication within some privileged sites (28).

Human immunodeficiency virus type 1 pol gene codes viral enzymes critical for viral replication. It is also the major drug target. Although the emergence of resistance mutations in the pol gene region associated with a reduced sensitivity to anti-retroviral drugs, those resistant mutations often result in the decreased catalytic activity and viral replicative capacity (79, 80). Whether the transmission and infections of drug resistant HIV-1 strain with reduced fitness lead to better disease outcome remains as a debatable topic, significantly lower viral load have been found in patients harboring M184V/I when compared to individuals carrying wild-type virus (Table 1) (29). Theoretically infection by viruses with impaired replicative capacity may have less serious impact to the hosts.

## The Effect of Different HIV-1 Subtypes and HIV-2 on Disease Progression Rates

Much of the understanding of disease progression derives from studies in HIV-1 subtype B strains. However, HIV-1 exhibits a high degree of inter- and intra-subtype genetic diversity (81). Such differences in the genetic characteristics of HIV not only play a role in the dynamics of HIV infection but also influence the biological properties including infectivity, transmissibility, and pathogenicity (82–85). Although there is no data supporting the infection of particular HIV-1 subtype with non-progressive HIV disease, individuals infected by subtype A appear to experience less risk of progression to death compare to non-A subtype (86, 87). In contrast, infection by HIV-1 subtype D has been shown to have a higher frequency of syncytium formation and X4 use, and consequently increased risk of progression to death (83, 85). Studies of HIV group O and HIV-2 also revealed significant reduced replicative and transmission fitness. This extremely low replicative capacity in comparison with that of HIV-1 group M strains has led to decreased group O and HIV-2 transmission and contributes to the low viral load and benign course of infection (88–90).

## Conclusion

In conclusion, study the viral factors in non-progression of HIV disease have provided great opportunities in understanding HIV gene functions and their contributions to viral pathogenesis. Genetic defects have been observed in many HIV-1 infected non-progressors. However, the lack of consistent pattern of genetic features in the LTNPs also suggest that control of HIV replication is not attributable to shared viral genetic defects or shared viral polymorphisms. In addition, it remains unclear how these defective mutations emerged initially and maintained in long-term in the LTNPs. Furthermore, many of the defective mutations are revertible and capable to evolve into virulent phenotype, hence the use of the defective virus as attenuated vaccine strains may not be completely safe. It is also worth to note that it is uncommon to discover defective virus from LTNPs and certain host characteristics need to be considered in the control of slowing disease process. A profound understanding of underlying host factors that force viral attenuation or defects to emerge in LTNPs, will provide new lead to HIV elimination and possible cure.

## Conflict of Interest Statement

The author declares that the research was conducted in the absence of any commercial or financial relationships that could be construed as a potential conflict of interest.
